# Supplementary material for: A Systematic Review and Developmental Perspective on Origin of CMS Genes in Crops
Source: Int J Mol Sci. 2024 Jul 31;25(15):8372. doi: 10.3390/ijms25158372 (PMC11312923; doi:10.3390/ijms25158372)
Supplement: Supplementary file 1 [file ijms-25-08372-s001.zip › ijms-3100241-supplementary.pdf]

Table S1. The different ten CMS sources lineage of Dian type hybrid rice.

**CMS sources of Dian type hybrid rice**	**Original maternal parent**	**Original paternal parent**	**Relationship between parents**
Dian type 1	*O. sativa L.* subsp. *Indica* at high altitude (Taibei 8 number)	*O. sativa* L. subsp *Japonica* at low altitude	Inter-subspecific
Dian type 2	*O. sativa* L. subsp. *Japonica* at high altitude (Mazaogu)	*O. sativa* L.subsp. *Indica* at high altitude (Eshandabaigu)	Inter-subspecific
Dian type 3	*O. sativa* L. subsp. *Indica* at high altitude (Eshandabaigu)	*O. sativa* L. subsp. *Japonica* at low altitude (Keqing 3 number)	Inter-subspecific
Dian type 4	*O. sativa* L. subsp. *Japonica* at high altitude (Zhaotongbeigu)	*O. sativa* L. subsp.*Japonica* at low altitude (Keqing 3 number)	Different ecotype
Dian type 5	*O. sativa* L. subsp. *Indica* at low altitude (Baotaiai)	*O. sativa* L. subsp. *Japonica* at high altitude (Hongmaoying)	Inter-subspecific
Dian type 6	*O. sativa* L. subsp. *Japonica* at low altitude (Keqing 3 number)	*O. sativa* L. subsp. *Japonica* at high altitude (Zhaotongbeigu)	Different ecotype
Dian type 7	*O. sativa* L. subsp *Indica* at high altitude (Indiaspringindica)	*O. sativa* L. subsp *Japonica* at low altitude (Keqing3number)	Inter-subspecific
Dian type 8	*O.sativa* L. subsp. *Japonica* at low altitude (Keqing three number)	*Oryza meyeriana* Baill． ssp． granulata Nees et Arn． ex Watt．(Shanlandao)	Inter-specific
Dian type 9	*O. rufipogon*	*O. sativa* L. subsp. *Japonica* (Nantezhan)	Inter-specific
Dian type 10	*Oryza meyeriana* Baill． ssp． granulata Nees et Arn． ex Watt．(Madenghong)	*O. sativa* L. subsp. *Japonica* (Heixuan 5 number)	Inter-specific
D type	*O. sativa L. subsp indica (Dissi)*	*O. sativa L. subsp indica*	Different ecotype
G type	*O. sativa L. subsp indica* (Gambiake kokum)	*O. sativa L. subsp indica (*Aijiaonante*)*	Different ecotype

Note: Variety names are in parenthesis. These materials were cited from the book entitled "Dian type hybrid rice memoir" (page 1-3) written by Li, Z.Y. and published by Yunnan people press (Kunming) in 1990.

Table S2. Twelve new CMS resources created by Rice Research Institute of Yunnan Agricultural University in recent years and theirs characteristics of pollen abortion.

		Average proportion of pollen of different abortion type			
Female parent	Generation and code name	Normal pollen	Uninucleate pollen abortion	binucleate pollen abortion	trinucleate pollen abortion
Diantun502	B1C1-1	5.37+4.7	5.45+2.6	63.80+10.0	25.38+7.6
D-7/4/IR28///IR28//Minghui63	B1C1-4	5.01+2.7	10.31+10.7	10.82+1.4	73.86+12.8
Yunhui290	B4C1-1	0.00+0.0	54.79+11.37	41.60+12.9	3.60+2.2
Nan7	B4C1-2	0.00+0.0	2.27+3.0	3.72+1.6	94.01+3.0
IR58025B	B4C1-4	0.00+0.0	60.53+7.3	38.24+6.0	1.24+1.3
IR58025B	B4C1-5	0.00+0.0	65.09+8.3	31.80+8.8	3.11+2.2
Miyang23	B4C1-6	0.00+0.0	62.89+6.8	30.18+5.6	6.93+6.3
Nan7-2	B4C1-8	0.00+0.0	5.28+3.0	10.33+5.3	84.39+8.2
Nanjing70507	B4C1-9	1.42+1.3	3.66+1.8	9.99+2.3	84.94+2.0
Nan34	B4C1-11	1.92+0.7	2.09+0.7	5.98+1.2	90.0+2.3
Minghui63	B5C1-1	0.00+0.0	7.22+4.1	6.48+1.8	80.78+2.9
Nanjing70507	B5C1-5	0.00+0.0	7.22+4.1	6.48+1.8	86.31+6.8

Note: These materials were cited from the conclusive report entitled as "Study on breeding techniques of Dian type Hybrid Rice and demonstration and Popularization of Dianza 31 and Dianza 32" in page 39-43.

As a famous soft conventional rice and a mid-early maturing indica rice, Diantun 502 was bred by Rice Research Institute of Yunnan Agricultural University and Gejiu seed Company in 1982, which still was planted in Yunnan province until now for its excellent quality. Minghui63 was the first excellent restorer line in the research of Chinese artificial grinding with strong resilience, broad-spectrum recovery and good combining ability. It has been widely applied in Chinese hybrid rice combination group and acquired good economic sense, which also mainly contributed to the breeding of new restorer lines as high-quality germplasm. IR58025, a conventional *indica* rice, was bred by International Rice Research Institute. Miyang23 is a conventional indica rice bred by Republic of Korea. Yunhui290 was a national conventional indica soft rice variety bred by Yunnan Academy of Agricultural Sciences and still widely planted until now. Nan34 was Dian type 1 and *japonica* type restorer line obtained from distant hybridization between *O. sativa* subsp. *indica* and *O. sativa* subsp. *japonica*. Nanjing70507 was bred by Jiangsu Academy of Agricultural Sciences as a conventional *indica* rice. In the creations of new CMS sources through distant hybridization between *O. sativa* subsp. *indica* and *O. sativa* subsp. *japonica, indica* type rice was usualy used as female parent.
